# The development of a novel medical education elective for fourth-year medical students

**DOI:** 10.5116/ijme.57f8.be5e

**Published:** 2016-10-22

**Authors:** Karima Khamisa, Suhair Bandeali, Ilan Fellus

**Affiliations:** 1 Division of Hematology, Faculty of Medicine, University of Ottawa, Canada; 2 Faculty of Medicine, University of Ottawa, Canada

**Keywords:** Development, novel medical education elective, fourth-year medical students, Canada

## Introduction

Postgraduate medical trainees (residents) have a well-established role in the teaching and training of undergraduate medical students across specialties.^1^ The need for these postgraduate trainees to be supported in their roles as key facilitators of undergraduate medical education has led to the mandatory inclusion of residents-as-teachers programs in all postgraduate residency training programs across Canada.^2^

In the United Kingdom, the National Health Service organizations are responsible for including a contractual requirement for doctors to carry out teaching.^3^ In the United States, the Accreditation Council for Graduate Medical Education (ACGME) Common Program Requirements for practice-based learning and improvement has outlined that residents/fellows are expected to develop skills and habits to be able to participate in the education of patients, families, students, residents and other health professionals, as documented by evaluations of a resident’s teaching abilities by faculty and/or learners.^4^ However, due to concurrent service requirements during residency, it is difficult for residents to devote time to enhancing teaching skills beyond the mandatory resident-as-teacher training program. Moreover, there is variability in the duration of such programs, with some devoting only a few hours to developing teaching skills.^5^^,^^6^

To enhance teaching confidence and abilities prior to residency, a number of institutions have developed programs for senior medical students to participate as mentors and near-peer instructors.^7^ Additionally, a number of medical schools in the United Kingdom and the United States have developed a formal curriculum for medical students to become junior clinical teachers.^8^^-^^12^

The transition from learner to teacher can conceivably be facilitated in the last year of medical school training. One prior study supports the use of this time to shape the professional identify of medical trainees.^13^ Without competing service requirements, an elective focused on teaching would be ideally placed in the fourth or final year of medical school training.  

In January 2015, the University of Ottawa introduced a clinical teaching elective (CTE) to fourth year medical students titled An Introduction to the Art and Science of Clinical Teaching. 

The purpose of this paper is to outline the development of this elective and its core curricular components.

### Curriculum design from conception to implementation

In identifying and exploring the objectives and content domains for the University of Ottawa Medical School CTE curriculum, we started with a systematic literature review.  It was interesting to note that no senior medical student electives were described in the literature from Canadian universities. We then reviewed the objectives for the resident-as-teachers course offered at our institution. Finally, we consulted with members of the core medical education research facility to further delineate goals and domains of knowledge to be acquired during the elective.

The two-week CTE was structured using Bloom’s taxonomy as a framework to organize educational objectives in sequential domains of learning theory and application.^14^   The CTE received approval by the University of Ottawa Faculty of Medicine Curriculum Content Review Committee (CCRC) in November 2014. The first cohort of 20 students completed the CTE in January 2015. To date, 50 students have completed the elective. Faculty members at the University of Ottawa with an interest in clinical teaching serve as volunteer lecturers and preceptors for the elective.   

### Curriculum components

#### Knowledge Comprehension

Students were exposed to basic principles in teaching and learning in medical education via lectures and self-directed readings. A hands-on workshop was offered during the elective on research tools, methods of dissemination, and medical education resources (including MedEdPORTAL). A librarian with expertise in medical education resources facilitated this session.

Residents are often expected to present current literature within meetings structured as a journal club. Within the elective, a workshop titled How to Conduct a Journal Club was facilitated by a clinician with expertise in epidemiology. A medical education article was appraised during this session.

#### Knowledge Application

Teaching, as with any acquired skill, requires deliberate practice and feedback from an experienced mentor to guide and improve technique.^15^

Participants were provided multiple opportunities to demonstrate their teaching skills in simulated and authentic settings. Participants led a bedside teaching session using both standardized and actual patients (consent was obtained in advance).  They also delivered lectures or presentations in an interactive and engaging manner to peers, and facilitated small group sessions for intra- and extra-curricular physical examination skills development for junior medical students. In addition, CTE participants served as examiners for a student-led formative clerkship OSCE (Observed Structured Clinical Examination).  Prior to being examiners, participants were given training on the principles of feedback and assessment in medical education. 

Mentorship is an important area of medical education and one of the twelve identified roles of a teacher, as described by Harden.^16^   Participants in the elective had the opportunity to serve as peer-mentors for first and second year medical students during an event titled Coffee with a Clerk. Junior medical students were invited to ask elective participants questions regarding any aspect of clerkship training or the residency matching process. 

#### Feedback and evaluation

Elective participants were given verbal feedback on their teaching skills by the elective supervisor, resident preceptors, peers and junior learners. While a number of structured teaching evaluation tools are available, we utilized a straightforward encounter tool for the purpose of written feedback.^17^

Reflection and self-assessment of teaching was encouraged throughout the elective. At the completion of the first offering of the elective, a focus group was conducted to prompt further reflection of the structure and content of the elective. The following year, participants were asked to complete an online exit survey with an option to provide free text statements to evaluate the program.  Studies will be needed to assess the impact of the elective on future educational praxis and skills of the CTE participants.

## Conclusions

The role of an educator is essential to the identity of all physicians and should be fostered and developed in the early stages of medical education.  The introduction of clinical teaching skills in the final year of medical school training is ideal given the lack of competing service requirements.  An elective in teaching must offer its participants a variety of learning modalities (lectures, workshops and authentic opportunities to mentor and teach junior colleagues).  The CTE at our institution has met with intense interest; 20-30 senior medical students have enrolled per year for the past three years.  This represents twenty percent of each class who have an interest in developing teaching skills. 

This novel elective aims to contribute to the training, growth and development of future clinical teachers and medical educators by providing hands-on teaching experiences, constructive personalized feedback, and evidence-based tools in clinical teaching. It is the most comprehensive elective of its kind for medical students interested in developing clinical teaching skills prior to residency.   In its current perception and structure, it can serve as a model for medical schools across North America.

### Conflict of Interest

The authors declare that they have no conflict of interest.
